# Design and Implementation of A CMOS Light Pulse Receiver Cell Array for Spatial Optical Communications

**DOI:** 10.3390/s110202056

**Published:** 2011-02-10

**Authors:** Md. Shakowat Zaman Sarker, Shinya Itoh, Moeta Hamai, Isamu Takai, Michinori Andoh, Keita Yasutomi, Shoji Kawahito

**Affiliations:** 1 Research Institute of Electronics, Shizuoka University, 3-5-1 Johoku, Nakaku, Hamamatsu, Shizuoka 432-8011, Japan; E-Mails: sarker@idl.rie.shizuoka.ac.jp (M.S.Z.S.); sito@idl.rie.shizuoka.ac.jp (S.I.); mhamai@idl.rie.shizuoka.ac.jp (M.H.); kyasu@idl.rie.shizuoka.ac.jp (K.Y.); 2 Toyota Central R&D Labs., Inc. 41-1 Yokomichi, Nagakute, Aichi 480-1192, Japan; E-Mails: takai@mosk.tytlabs.co.jp (I.T.); salimsarker@gmail.com (M.A.)

**Keywords:** spatial optical communication, CMOS image sensor, LED, light pulse receiver

## Abstract

A CMOS light pulse receiver (LPR) cell for spatial optical communications is designed and evaluated by device simulations and a prototype chip implementation. The LPR cell consists of a pinned photodiode and four transistors. It works under sub-threshold region of a MOS transistor and the source terminal voltage which responds to the logarithm of the photo current are read out with a source follower circuit. For finding the position of the light spot on the focal plane, an image pixel array is embedded on the same plane of the LPR cell array. A prototype chip with 640 × 240 image pixels and 640 × 240 LPR cells is implemented with 0.18 μm CMOS technology. A proposed model of the transient response of the LPR cell agrees with the result of the device simulations and measurements. Both imaging at 60 fps and optical communication at the carrier frequency of 1 MHz are successfully performed. The measured signal amplitude and the calculation results of photocurrents show that the spatial optical communication up to 100 m is feasible using a 10 × 10 LED array.

## 1. Introduction

Light emitting diodes (LEDs) have been widely used for various applications such as illumination, display, traffic lights, head and tail lights of automobiles. Unlike other light sources, the LED can switch at relatively high speed. The wide spreading application of LEDs allows us to open up a new era of spatial optical communications [1–3].

The image sensor communication (ISC) technology is essential for the spatial optical communication because to find the light source and to intensify the light energy density at the receiver, the optical receiver has to have a photo detector array and signal light source finding and tracking functions. A few approaches for ISC have been reported. CMOS ISC chips have been used for ID beacon detection [4,5], where very low data rates are required. In an ISC chip for an optical wireless LAN application [6], the data rate of several hundreds of MHz has been demonstrated. In this approach, however, photo-diode current of a 2-D detector array directly flows into external receiver circuits, and because of this, extremely large optical power using laser lights is required.

In this paper, a CMOS optical light pulse receiver (LPR) cell array with an imager on the same plane is presented. The main target application is car-to-car and road-to-car spatial optical communications. The purpose of this development is to achieve long-range spatial optical communication up to 100 m using cheap LED light sources, moderate data rate up to 1 MHz, and a light-source finding and tracking function. Toward this goal, the proposed architecture uses a mixed highly-sensitive CMOS imager and wide-dynamic-range light pulse receiver array for the received optical pulses based on a pinned photodiode technology [7,8]. A successful spatial optical communication is demonstrated using a developed VGA-size prototype chip. This paper focuses on the design and evaluation of the CMOS LPR cell array with the theory for the transient response, device simulations, and chip implementation. The ISP chip is designed for moving target light source finding and tracking. The image signal output and 25-channel parallel LPR cell signal outputs can be used for this purpose. In the experiment of this paper, however, the image signal is used for finding non-moving light source and one-channel LPR cell signal output only is used for the demonstration of the spatial optical communication.

## 2. Chip Architecture

### 2.1. Image-Sensor-Based Optical Communication Systems

**Figure 1.**
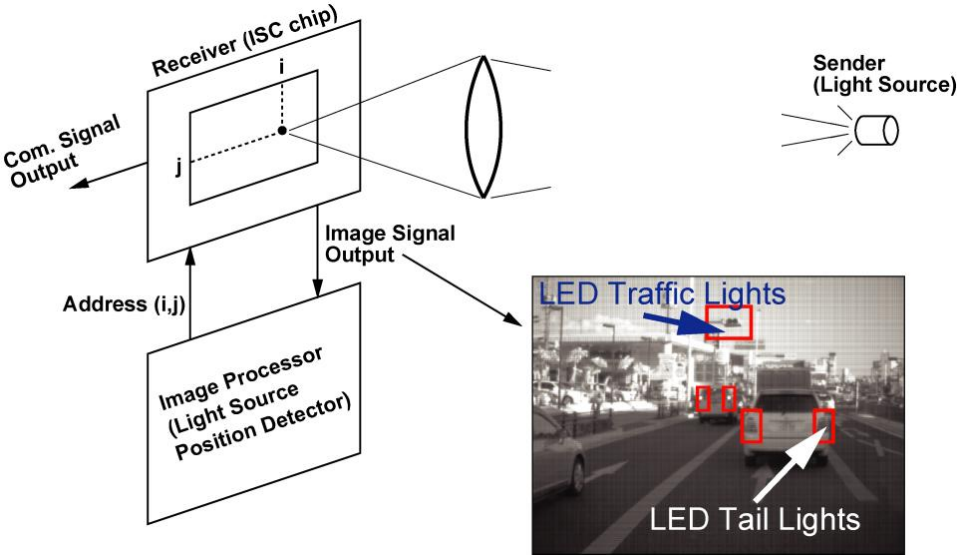
Image-Sensor-Based Optical Communication System.

A conceptual figure of the proposed image-sensor-based optical communication systems is depicted in Figure 1 [7]. In this system, a CMOS sensor which has both an imaging pixel array and LPR cell array is used. The lens of the ISC camera focuses the light sources at the focal plane of the CMOS sensor. This greatly enhances the optical energy density at the focal plane and by receiving the optical energy with a small-size photodiode, relatively high-speed optical communication is realized if the position of the signal spot lights irradiation on the focal plane is known. Using an image signal output, an external image processor finds the position of the signal spot light and the vertical address (V-address) and horizontal address (H-address) are sent back to the CMOS sensor chip. Then the communication signals from the selected address and its peripherals are read out.

### 2.2. ISC Chip Architecture and Circuits

**Figure 2.**
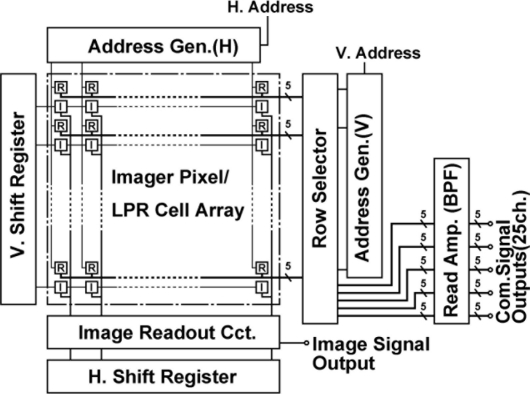
Architecture of the CMOS ISC chip (R: LPR cell, I: Image Pixel).

Figure 2 shows the block diagram of the ISC chip. It consists of imaging pixel and LPR cell arrays, readout circuits for image signals including Vertical (V.) and Horizontal (H.) shift registers and readout circuits for communication signals including V- and H- address generators, analog multiplexer, and bandpass readout amplifiers.

**Figure 3.**
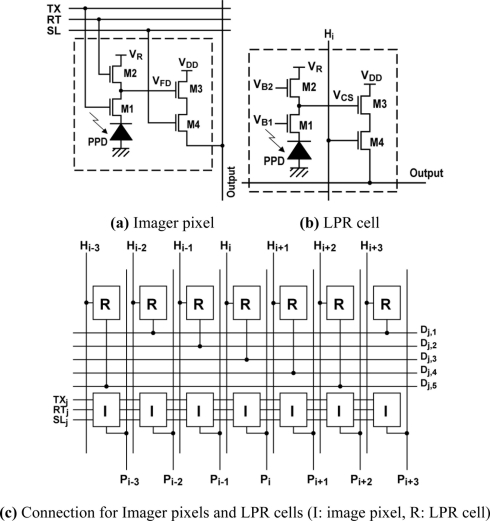
Imager Pixel and LPR Cells.

**Figure 4.**
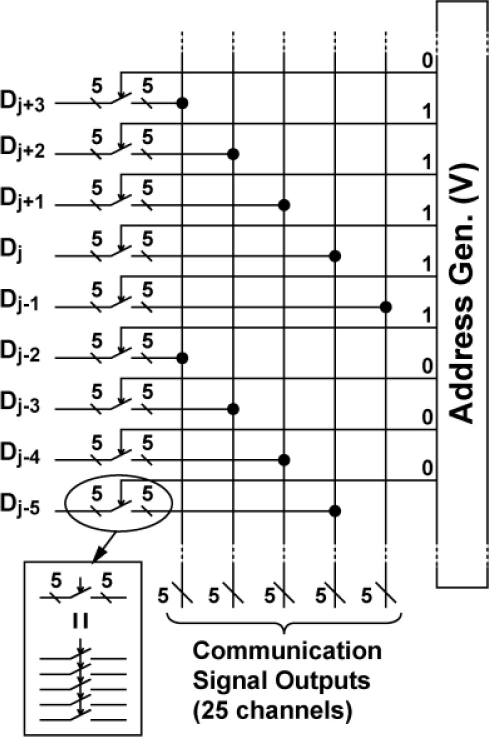
Row selector for Communication Signal Bus.

**Figure 5.**
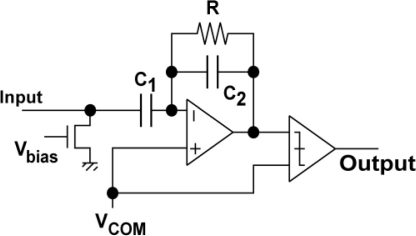
Bandpass amplifier and comparator.

Figures 3(a,b) show the imager pixel and LPR cell, respectively. Both the pixel and LPR cell use four transistor circuits. In the LPR cell, the gate of transistors M_1_ and M_2_ are connected to constant bias sources. To respond to the light pulse charge, the transistor M1 is working in sub-threshold region. The detailed operation of the LPR cell is described in the next section. The operation of the imager pixel is same as that of the conventional 4-transistor active pixel with a pinned diode [9,10], which is controlled by signals, SL, RT and TX for pixel selection, resetting floating diffusion (FD) amplifiers and charge transfer from a photodiode to the FD, respectively. The detailed operation of the CMOS image sensors using pinned photodiodes is described, for instance, in [11]. The connections of control signals and outputs for the imager pixel and LPR cells are shown in Figure 3(c). The imager pixels and LPR cells are assigned to odd and even rows, respectively. In order to read out 5 × 5 LPR cell outputs at the same time, every five horizontal pixels are connected to five horizontal signal lines. If the *i*-th LPR cell is the center, the five LPR cells are selected by signals *H_i−2_*, *H_i−1_*, *H_i_*, *H_i+1_*, and *H_i+2_* from the H-address generator. The outputs are connected to a row selector as shown in Figure 4, where five row outputs of the LPR cells, D_j−2_, D_j−1_, D_j_, D_j+1_ and D_j+2_, are selected by the V-address generator. To do this, the outputs of the V-address generator are activated to make the bus switches for LPR cell outputs, D_j−2_, D_j−1_, D_j_, D_j+1_ and D_j+2_ on as shown in Figure 4. The selected 5 × 5 or 25-channel LPR cell outputs are connected to 25-channel bandpass amplifiers whose circuit schematic of one channel is shown in Figure 5.

**Figure 6.**
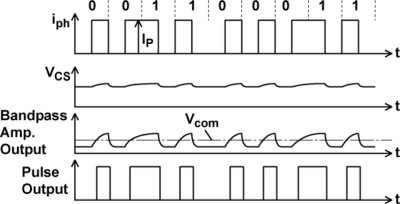
Waveforms in a readout channel.

**Figure 7.**
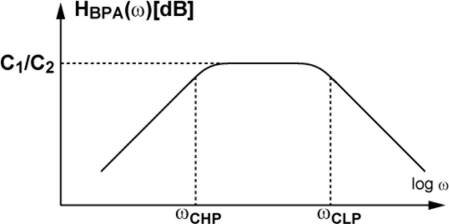
Frequency Response of Bandpass Amplifier.

The waveforms of each stage of a readout channel are shown in Figure 6. At the input, a load current source for a source follower is connected. The in-pixel transistor M3 in Figure 3(b) and the current source comprise a source follower when M4 is turned on. The output is amplified by a bandpass amplifier whose frequency response is shown in Figure 7. The source follower has a large offset deviation mainly due to the threshold voltage variation of M3. This may disturb the detection of the LPR signal of small amplitude if the source follower output including DC components is directly amplified. The input capacitor C_1_ of the bandpass amplifier cuts the DC component of the input signal and the resulting small AC signal modulated for optical communication using, e.g., Manchester coding, is amplified by the gain given by the capacitor ratio, C_1_/C_2_. In the bandpass amplifier, the high-pass cut-off frequency f_CHP_ in Figure 7 is given by 1/2πRC_2_. The high-pass cut-off frequency has to be sufficiently lower than the carrier frequency to be used for spatial optical communication in order to pass the lower sideband of the modulated signals. For testing the designed ISC chip at the carrier frequency of 100 kHz to 1 MHz, the cutoff frequency is chosen as a few kHz. The low-pass cut-off frequency f_CLP_ is determined by the bandwidth of the internal opamp and is given by g_m_/2πC_1_, where g_m_ is the transconductance of the CMOS internal operational transconductance amplifier (OTA). The low-pass cut-off frequency must be sufficiently higher than the carrier frequency to be tested and is chosen as about 10 MHz. The amplified signal is digitized with a comparator to produce a pulse signal output. This approach is useful for the simplification of the total system because the external system can be implemented with digital circuits and software. On the other hand, for a long distance communication with weak optical signals, the analog waveforms of the amplifier outputs are digitized with high-sampling rate A/D converters and a digital equalizer should be applied for a better eye opening [12].

However, for 25-channel outputs necessary for the light source tracking, 25-channel A/D converters are necessary in the external system which results in a bulky system and large cost. The threshold voltage of the comparator is chosen as V_COM_ so that the bandpass amplifier output is digitized at the zero-crossing point of the waveform.

### 2.3. Light Spot Position Detection

**Figure 8.**
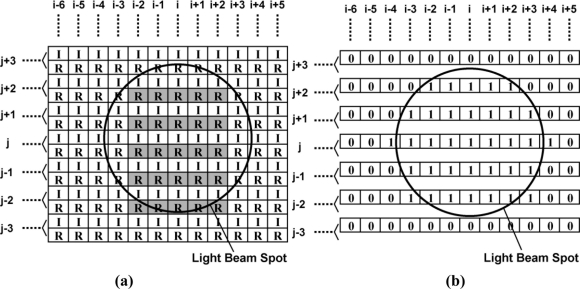
**(a)** Light beam spot and **(b)** Binary image of the light beam spot.

To find the position of the center of the light beam spot for optical communication, the image output can be used. If a LED light beam spot irradiates the focal plane as shown in Figure 8(a), the position of the center pixel of the light beam spot must be (i, j). As described in the next section, the photo current intensity due to the direct LED light beam spot for communication is much larger than that induced by the standard illumination level. Therefore, by choosing a very small accumulation time, the signal level of the image other than the region of light beam spot becomes very small and the resulting image contains light beam spot only. The region of the light beam spot can be extracted as shown in Figure 8(b) using a binarization algorithm given by:

(1) $$G(x,y)=\{\begin{array}{ll} 1 & \left(\text{if }f(x,y)\geq T\right)\\ 0 & \left(\text{if }f(x,y)<T\right) \end{array}(\text{for x}=1,...,\;N,\;y=1,...,M)$$

where f(x,y) is the input gray-scale image at (x,y), G(x,y) is the binary image at (x,y), T is the threshold of binarization, N is the number of horizontal pixels, M is the number of vertical pixels. To know the address of the center pixel, an algorithm to find the center-of-gravity given by:

(2) $$i=\sum_{x=1}^{N}\sum_{y=1}^{M}\mathrm{xG}(x,y)/\sum_{x=1}^{N}\sum_{y=1}^{M}G(x,y)$$

(3) $$j=\sum_{x=1}^{N}\sum_{y=1}^{M}\mathrm{yG}(x,y)/\sum_{x=1}^{N}\sum_{y=1}^{M}G(x,y)$$

**Figure 9.**
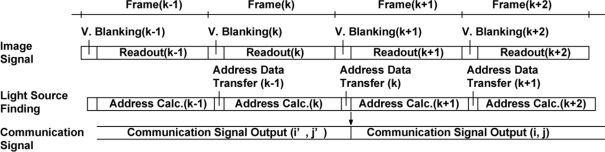
Timing diagram of the image signal readout, light source address calculation and communication signal output.

If the addresses *i* and *j* are not an integer, they are rounded to the nearest integer. The addresses *i* and *j* are sent to the address decoders for the vertical and horizontal directions, respectively, and then the 5 × 5 outputs of the *i* − 2 through *i* + 2 cells for horizontal direction and the *j* − 2 through *j* + 2 cells for vertical direction as shown in the shaded R cells in Figure 8(a) are selected and connected to the 25-channel bandpass amplifiers. The 25-channel parallel outputs can be used for tracking a moving target light source. Figure 9 shows a typical timing for light source tracking. While reading the image signal of the *k*-th frame, the V- and H- addresses of the center of the light beam slot are calculated. In the Vertical blanking time, or right after finishing the image signal readout of the *k*-th frame, the address data of the *k*-th frame are transferred to the ISC chip. If the V- and H- addresses of the *k*-th frame is different from that of the (*k-1*)-th frame, the V- and H- addresses for the address decoder in the ISC chip is renewed. Then the communication signals are readout from the new position.

## 3. LPR Cell Design

### 3.1. LPR Cell Operation

**Figure 10.**
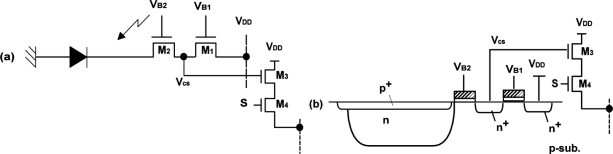
**(a)** LPR cell **(b)** Cross section of LPR cell.

Figure 10(b) shows the simplified cross-section of the LPR cell whose schematic correspondence is shown in Figure 10(a). A pinned photodiode (PPD) is used for photo detection. The charge sensing node (voltage: V_CS_) is coupled to the PPD through M_2_ and a drain (n^+^) through M_1_. The drain terminal is connected to a power supply voltage V_DD_. The gates of M_1_ and M_2_ are biased to V_B1_ and V_B2_, respectively. The charge sensing node is connected to the gate of transistor M_3_ which is used for a source flower readout amplifier. A transistor M_4_ is used for the selection of the pixel.

**Figure 11.**
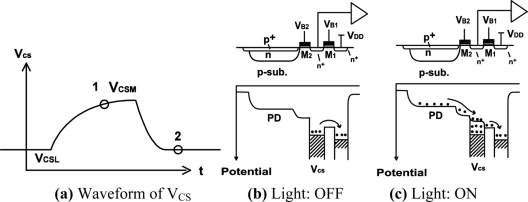
Operation of LPR Cell.

Figure 11 shows the operation of the LPR cell with potential profiles. The voltage V_B2_ for M_2_ is biased such that the pinned diode is always depleted if there is no light. A transistor M_1_ is biased to V_B1_ and it is higher than V_B2_. If a light pulse irradiates the photodiode, a photo-generated charge is rapidly transferred to the charge sensing node, and a part of the charge is drained to V_DD_ through M_1_. Since M_1_ is working at sub-threshold region, the V_CS_ responds to the logarithm of the photo current and the V_CS_ is going down when light is on. When light is off, the excess electron in the floating diffusion is drained to V_DD_, and the voltage is going up. The turning-off response time dominated by diffusion current is much larger than that of the turning-on.

### 3.2. Transient Response

**Figure 12.**
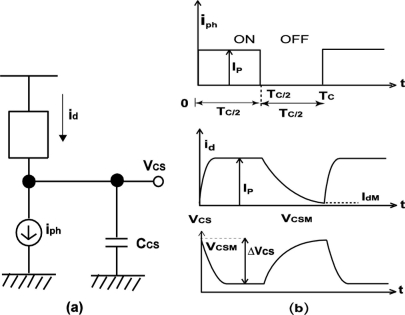
**(a)** Model of transient analysis of LPR cell, **(b)** Waveform of I_ph_, i_d_ and V_CS._

The transient response of the LPR cell is analyzed using an equivalent circuit of Figure 12(a) for the LPR cell. The sub-threshold current *I_d_* flowing through transistor M_1_ is an exponential function of the gate-source voltage of M_1_, *V_B1_−V_CS_*, and it is given by:

(4) $$I_{d}=I_{d0}\;\text{exp}\left\{\frac{1}{{\mathrm{nV}}_{\mathrm{th}}}\left(V_{B1}-V_{\mathrm{CS}}-V_{T}\right)\right\}$$

where *V_T_* is threshold voltage, *I_d0_* is the current if *V_B1_* − *V_CS_* = *V_T_*, *n* is an ideality factor, and *V_th_* = *k_B_T/q* is a thermal voltage.

When the light is on, the drain current *I_d_* increases up to the photocurrent *I_P_* at *t* = *T_C_/2*, where *T_C_* is the cycle time of the light pulse. When the light is off, the drain current decreases, finally to *I_dM_* at *t* = *T_C_*. The charge sensing node voltage at *t* = 0 is denoted by *V_CSM_*. Then the drain current can be written as:

(5) $$I_{d}=I_{dM}\;\text{exp}\left\{\frac{-1}{{\mathrm{nV}}_{\mathrm{th}}}\left(V_{\mathrm{CS}}-V_{\mathrm{CSM}}\right)\right\}$$

From Figure 12(a), the drain current can be written as:

(6) $$I_{d}=\left\{\begin{array}{cc} C_{\mathrm{CS}}\;\frac{{\mathrm{dV}}_{\mathrm{CS}}}{\mathrm{dt}}+I_{P} & \left(0<t\leq T_{C}/2\right)\\ C_{\mathrm{CS}}\;\frac{{\mathrm{dV}}_{\mathrm{CS}}}{\mathrm{dt}} & \left(T_{C}/2<t\leq T_{C}\right) \end{array}\right\}$$

In Figure 12(a), the influence of the photodiode capacitance is ignored and the photo charge generated in the photodiode is instantly transferred to the charge sensing node.

By solving Equation (6) with Equation (5) and initial conditions for turning light on and off, the drain currents during turning on, *I_d,on_* and turning off, *I_d,off_* are given by:

(7) $$I_{d,\mathrm{on}}(t)=\frac{I_{P}}{1+\left(\frac{I_{P}}{I_{\mathrm{dM}}}-1\right)\;\text{exp}\;\left(\frac{t}{\tau }\right)}$$

(8) $$I_{d,\mathrm{off}}(t)=\frac{I_{P}}{1+\left(t-\frac{T_{C}}{2}\right)/\tau }$$

where τ is a time constant given by:

(9) $$\tau =\frac{{\mathrm{nV}}_{\mathrm{th}}C_{\mathrm{CS}}}{I_{p}}$$

Since *I_d, off_* (T_C_) = *I_dM_*, Equation (8) gives:

(10) $$\frac{I_{P}}{I_{\mathrm{dM}}}=1+\frac{T_{C}}{2\tau }=1+\frac{1}{2\tau f}$$

where *f* = *1/T_C_* is the pulse frequency.

From Equations (5), (7) and (8), for 0 < *t* ≤ *T_c_*/2:

(11) $$\begin{array}{l} V_{\mathrm{CSM}}-V_{\mathrm{CS}}(t)={\mathrm{nV}}_{\mathrm{th}}\;\text{ln}\frac{I_{d,\mathrm{on}}(t)}{I_{\mathrm{dM}}}\\ ={\mathrm{nV}}_{\mathrm{th}}\;\text{ln}\frac{I_{P}}{I_{\mathrm{dM}}}\frac{1}{1+\left(\frac{I_{P}}{I_{\mathrm{dM}}}-1\right)\;\text{exp}\;\left(-\frac{t}{\tau }\right)} \end{array}$$

and for *T_c_*/2 < *t* ≤ *T_c_*:

(12) $$\begin{array}{l} V_{\mathrm{CSM}}-V_{\mathrm{CS}}(t)={\mathrm{nV}}_{\mathrm{th}}\;\text{ln}\frac{I_{d,\mathrm{off}}(t)}{I_{\mathrm{dM}}}\\ ={\mathrm{nV}}_{\mathrm{th}}\;\text{ln}\frac{I_{P}}{I_{\mathrm{dM}}}\frac{1}{1+(t-T_{C}/2)/\tau } \end{array}$$

The signal amplitude, Δ*V_CS_* is then given by:

(13) $$\begin{array}{l} \;\;\;\Delta V_{\mathrm{CS}}=V_{\mathrm{CSM}}-V_{\mathrm{CS}}\;\left(T_{C}/2\right)\\ ={\mathrm{nV}}_{\mathrm{th}}\;\text{ln}\frac{I_{P}}{I_{\mathrm{dM}}}\frac{1}{1+\left(\frac{I_{P}}{I_{\mathrm{dM}}}-1\right)\;\text{exp}\;\left(-\frac{1}{2\;f\;\tau }\right)} \end{array}$$

Since *V_CM_* − *V_CS_* (*T_C_*) = 0, the following relationship has to be met from Equation (12).

(14) $$\frac{I_{P}}{I_{\mathrm{dM}}}=1+\frac{1}{2\tau \;f}$$

From Equations (10), (13) and (14), Δ*V_CS_* is finally given by:

(15) $$\Delta V_{\mathrm{CS}}={\mathrm{nV}}_{\mathrm{th}}\;\text{ln}\frac{1+\frac{1}{2\tau \;f}}{1+\frac{1}{2\tau \;f}\;\text{exp}\;\left(-\frac{1}{2\;f\;\tau }\right)}$$

(16) $$\begin{array}{c} ≅{\mathrm{nV}}_{\mathrm{th}}\;\text{ln}\frac{1}{2\tau \;f}\;(\text{if}\;2\tau \;f\;□\;\;1)\\ ={\mathrm{nV}}_{\mathrm{th}}\;\text{ln}\frac{f_{c}}{f} \end{array}$$

where *f_c_* = 1/2*τ* is a cutoff frequency of the charge sensing node. In the frequency range *f* << *f_c_*, the signal amplitude logarithmically deceases to the pulse frequency. This property is useful for wide working frequency range of the optical pulse receiver. For the frequency range *f* > *f_c_*, the amplitude rapidly decreases to zero. For example, if *f* = 10*f_c_*, Δ*V_CS_* ≅ 0.01*nV_th_*. Therefore, the design to have a small *τ* is very important for the wideband receiver. Obviously a smaller charge sensing capacitance and large photo current are necessary for the wideband receiver.

## 4. Simulations

### 4.1. Photo Current as a Function of Distance

Since the response of the receiver directly depends on the photo current level, the estimation of the available photo current level generated by the photodiode in a pixel in the actual communication system is necessary.

Photo current generated in photodiode with the area of *A_PD_*[*μm*^2^] and the quantum efficiency of η*_Q_* is given by:

(17) $$I_{P}={q\eta }_{Q}\frac{A_{\mathrm{PD}}}{E_{P}}p_{0}$$

where *E_p_* is the photon energy and *p*_0_ is the optical power per unit area at the focal plane. The radiant flux F at the lens is given by:

(18) F=∫Idω =Iω

if *I* is supported to be constant at the lens area, where *I* is the radiant intensity of LEDs and ω is the solid angle given by:

(19) $$\omega =\frac{S_{L}}{L^{2}}=\frac{\pi {\left(\frac{D}{2}\right)}^{2}}{L^{2}}$$

where *S_L_* is the area of the lens, *L* is the distance to the LED and D is the diameter of the lens. The relationship between the area of the LED array *S_LED_* and the image size at the focal plane *S_F_* is given by:

(20) $$\frac{S_{F}}{S_{\mathrm{LED}}}=\frac{f_{l}^{2}}{L^{2}}$$

where *f_l_* is the focal length.

The optical power per unit area at the focal plane is given by:

(21) $$p_{0}=\{\begin{array}{cc} \frac{{\mathrm{FT}}_{L}}{S_{F}} & \left(\mathrm{if}\;S_{F}>S_{P}\right)\\ \frac{{\mathrm{FT}}_{L}}{S_{P}} & \left(\mathrm{if}\;S_{F}\leq S_{P}\right) \end{array}$$

where *T_L_* is the transmittance of the lens and *S_P_* is the area of pixel.

For *S_F_* > *S_P_*, *p*_0_ is given by:

(22) $$p_{0}=\frac{\pi T_{L}I}{4F_{N}{}^{2}S_{\mathrm{LED}}}$$

where *F_N_*(= *f_l_*/*D*) is the F number of the lens.Therefore *p*_0_ becomes constant independent of the distance to the LED.

For *S_F_* ≤ *S_P_*, *p*_0_ is given by:

(23) $$p_{0}=\frac{{\mathrm{IT}}_{L}}{L^{2}}.\frac{S_{L}}{S_{P}}$$

Therefore, for the distance larger than $f_{l}\sqrt{S_{\mathrm{LED}}/S_{P}}$, the optical power is reduced inversely proportional to the square of the distance.

**Figure 13.**
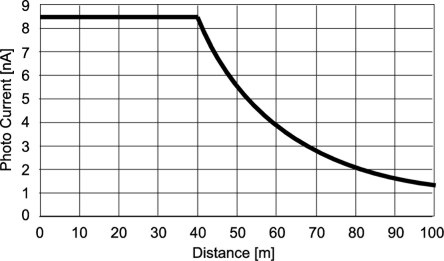
Photo current verses distance (LED Array size: 5 cm × 5 cm, LED: λ = 870 nm, IE = 80 mW/sr, 10 × 10 array, cell size: 7.5 μm × 7.5 μm, η = 24%@870 nm, Lens: FN = 1.4, TL = 0.75).

Figure 13 is a calculation result of photo current generated in 7.5 μm × 7.5 μm size pixel as a function of the communication distance. In this result, photo current is constant up to 40 m, and it is larger than 8 nA. It is reduced inversely proportional to L^2^ for the range larger than 40 m but it is still larger than 1 nA at 100 m. This current level of nA order is sufficient for obtaining large voltage swing at the sensing node of the LPR cell for the pulse frequency of 1 MHz.

### 4.2. Device Simulations

**Figure 14.**
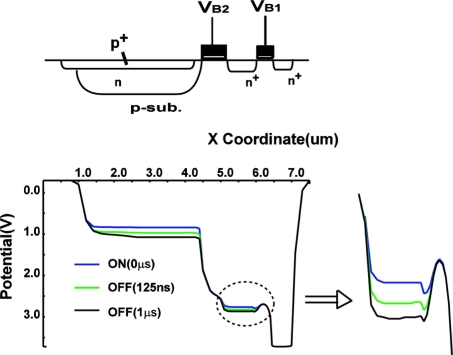
Potential profile during turning on and off.

Device simulations have been conducted to measure the response of the charge sensing node to the light pulse. Figure 14 shows simulated potential profiles for turning on (125 ns and 1 μs from the beginning when the light is on) and off. Both potentials of photodiode and charge sensing node are going up for turning off and going down for turning on. The response of the photodiode potential is not so much different from that of the charge sensing node. Therefore, the capacitance of the photodiode influences to the response while the model of the transient analysis in Section **3.2** considers the charge sensing node capacitance only. The influence of the photodiode capacitance can be translated into the increase of the equivalent charge sensing node capacitance. Figure 14(a,b) show the simulated waveform of photocurrent and voltage response of V_CS_ node in light on and off conditions. For the turning-on response, *y_on_*(*t*) defined as follows is introduced.

(24) $$y_{\mathrm{on}}(t)=\text{ln}\left\{\frac{I_{P}}{I_{d,\mathrm{on}}(t)}-1\right\}$$

Using Equation (7), *y_on_*(*t*) is given by:

(25) $$y_{\mathrm{on}}(t)=\text{ln}\left\{\frac{I_{P}}{I_{\mathrm{dM}}}-1\right\}-\frac{t}{\tau }$$

Therefore, *y_on_*(*t*) is a linear function of *t* and the time constant *τ* can be obtained by the gradient of the plot of *y_on_*(*t*).

For the turning-off response, *y_off_*(*t*) defined as follows is introduced:

(26) $$y_{\mathrm{off}}(t)=\frac{I_{P}}{I_{d,\mathrm{off}}(t)}$$

Using Equation (11), *y_off_*(*t*) is expressed as:

(27) $$y_{\mathrm{off}}(t)=1+\left(t-T_{C}/2\right)/\tau$$

Therefore, *y_off_*(*t*) is also a linear function of *t* and the time constant *τ* can also be obtained by the gradient of the plot of *y_off_*(*t*).

**Figure 15.**
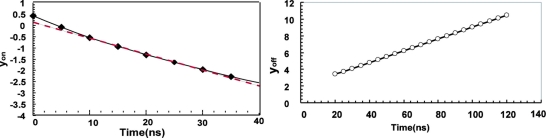
Measured of time constant *τ* from simulation result **(a)** Light ON phase and **(b)** Light OFF phase.

**Figure 16.**
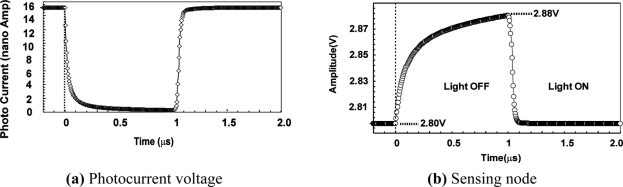
Simulated waveforms.

Figure 15(a,b) are plots of *y_on_*(*t*) and *y_off_*(*t*), respectively, which are calculated from the simulation results of Figure 16(a,b). As shown in Figure 16, both *y_on_*(*t*) and *y_off_*(*t*) are approximately linear functions of *t*. The summary of the simulation results is shown in Table 1. The time constants for turning on and turning off calculated by the gradient of the plots are 14.0 ns and 14.2 ns, respectively. Since the plot of y_on_ has a slight non-linearity, the time constant is calculated by the time range of 10 to 30 ns. From these simulation results, the charge sensing node capacitances for turning on and turning off are 6.8 fF and 6.9 fF, respectively.

### 4.3. Circuit Simulation of the Readout Circuits

**Figure 17.**
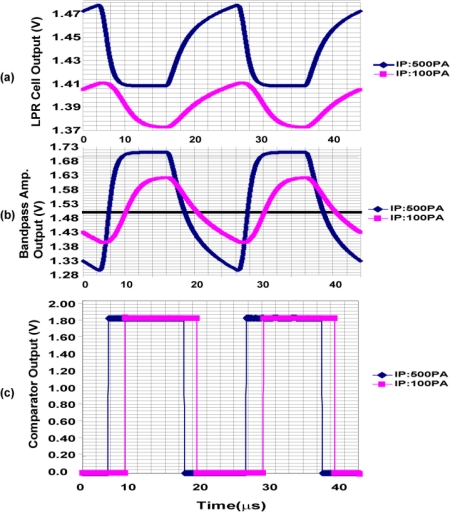
Simulated waveforms of the LPR cell, bandpass amplifier and comparator outputs.

Figure 17 shows simulated waveforms of LPR cell source follower output, readout amplifier output and pulsed (digital) output using a circuit simulator SPECTRE. Results for two photo current levels of 100 pA and 500 pA are shown.

The DC offset levels at the charge sensing node for the two signals with different photo current levels are purposely changed and the resulting source follower outputs of the two input levels have different DC offset levels. At the output of the bandpass amplifier, however, the two signals have the same offset level of V_COM_ (= 1.5 V) because the input capacitor C_1_ does not pass the DC offset of the source follower output. This high-pass filtering function of the amplifier is helpful for receiving signals with small amplitude despite the charge sensing node and the source follower have a large offset deviations. Circuit parameters in Figure 6 are C_2_ = 3.8 pF, C_1_ = 22.8 pF, R = 20 MΩ and g_ma_ = 1.37E − 3 [1/Ω] and the resulting high-pass and low-pass cutoff frequencies are 2 kHz and 9.6 MHz, respectively. The pass-band gain is 15.5 dB which is identical to the capacitance ratio of C_1_/C_2_ = 6.

## 5. Experimental and Results

**Figure 18.**
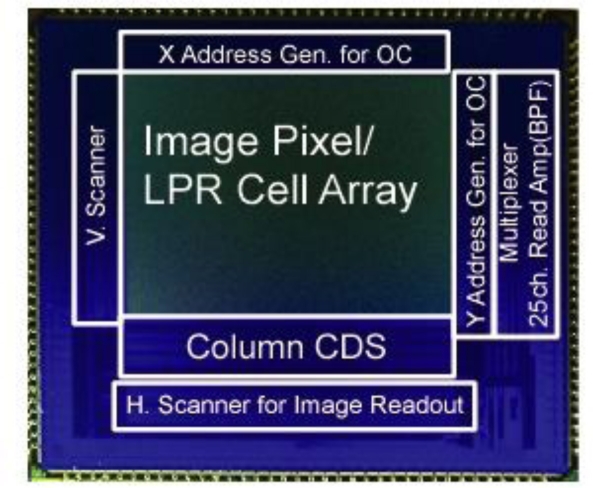
The micrograph of CMOS image sensor chip.

A CMOS sensor for imaging and spatial optical communication is designed and implemented using 0.18 μm CIS technology. Figure 18 shows a microphotograph of the chip. It has 640 × 240 pixel image array and 640 × 240 LPR cell array with analog/digital readout circuits. The pixel and LPR cell occupy 7.5 × 7.5 μm^2^ with 20% fill factor. The parameters of the fabricated sensor are summarized in Table 2.

**Figure 19.**
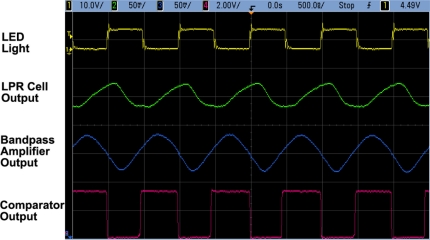
Waveformes of communication signals.

Figure 19 shows typical waveforms of the communication signal outputs observed using test channel outputs. The communication cell outputs (pixel source follower output, readout amplifier output and pulsed (digital) output) are successfully read out at the frequency of 1 MHz and photo current amplitude of 5 nA.

**Figure 20.**
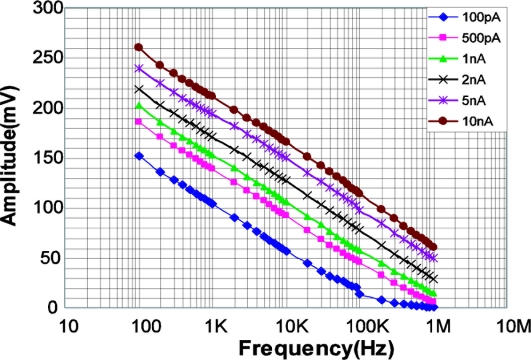
Amplitude as a function of Pulse Frequency (LED: λ = 870 nm, IE = 80 mW/sr, Lens: FN = 1.4, f = 6 mm, Distance: 2 m).

Figure 20 shows the measurement results of signal amplitude of the LPR cell output (source follower output) as a function of pulse frequency. As explained in the analysis of the pixel response, the amplitude has a linear response to the logarithm of the frequency. At the frequency of 1 MHz, amplitude of more than 10 mV is obtained for the photocurrent of 1 nA.

**Figure 21.**
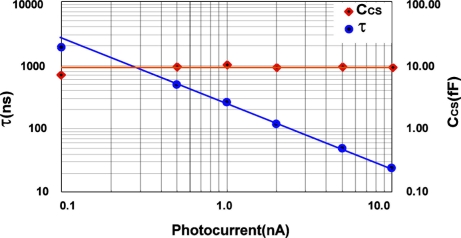
Estimated time constant and charge sensing capacitance as a function of photo current.

From Figure 20, Equation (9), and Equation (16), the time constant and sensing node capacitance are estimated as a function of photo current as shown in Figure 21. The time constant is inversely proportional to the photocurrent as predicted by Equation (9). The sensing node capacitance is almost constant at around 9 fF to the photocurrent except for very low photocurrent of 100 pA at which a measurement error due to the poor SNR occurs. This capacitance is larger than the value estimated by the device simulations, where C_CS_= 6.8–6.9 fF. The reason of the difference between the simulation and the experimental result is mainly due to capacitance of the source flower transistor and wiring.

**Figure 22.**
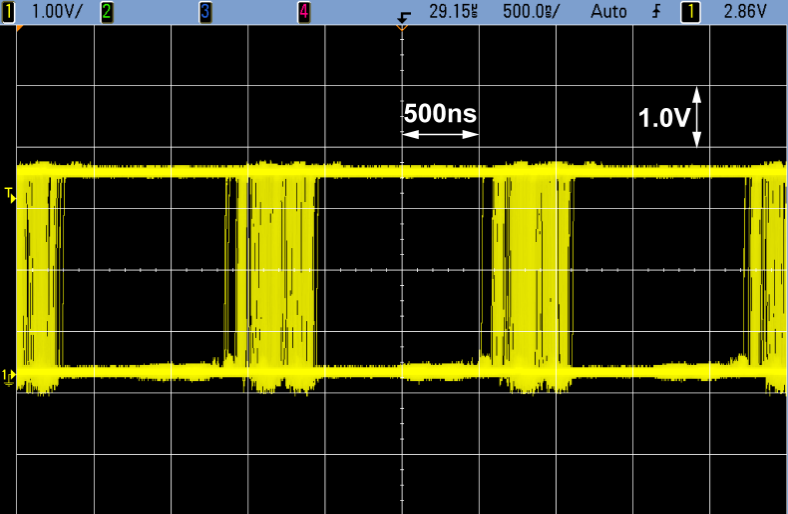
Eye Diagram.

Figure 22 shows an eye diagram of the communication signal output at the carrier frequency of 300 kHz and the photo current level of 2.2 nA. The received optical signal is modulated by random baseband signal and Manchester coding. At this frequency and photo current level, a sufficiently large eye opening is realized.

**Figure 23.**
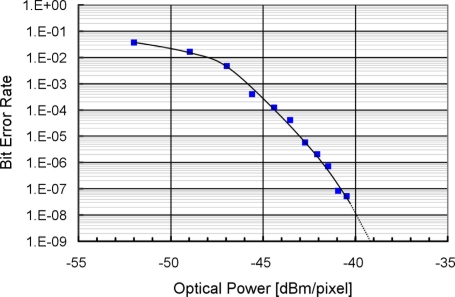
Bit Error Rate.

Figure 23 shows the BER (Bit Error Rate) as a function of optical power measured at 500 kbps. An acceptable BER for spatial optical communication is larger than 1E-5, which corresponds to the optical power per pixel of 50 nW. With the measured radiant sensitivity of the photo diode of 0.07 A/W at 870 nm, the photo current level is 3.5 nA. From Figure 13, this photo current level is available at the communication distance of 65 m. The same signal voltage amplitude with a smaller photo current level can be obtained if the communication data rate is slower. Theoretically, the photo current level of 1.3 nA which corresponds to the available photo current level at 100 m provides the same signal voltage amplitude at the data rate of 186 kbps from Equations (9) and (16).

**Figure 24.**
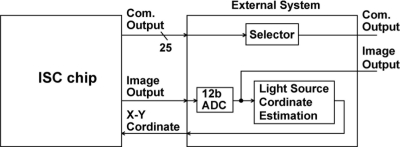
Setup for image data transmission.

As a basic testing of the implemented ISC chip, a spatial optical communication between non-moving object (sender: a LED light source) and the non-moving camera (receiver: ISC chip) has been carried out. Figure 24 shows an experimental setup for image data transmission. In this experiment, a scene is captured by a CCD camera, the image is modulated by a Manchester code, and LED array (4 × 4) is driven by the modulated signal of the image. An image with driving LED array is taken by the implemented CMOS sensor. The distance from the LED array to the CMOS sensor is set to 3 m. Since no tracking method is implemented in this experiment, the treatment of 25-channel outputs is simplified. A signal of one channel at the center of 25 LPR cells is selected. In this experiment, the horizontal and vertical addresses in the LPR cell array that produces the largest signal amplitude are estimated manually by a cut-and-try method. The LPR output is sent to a PC to reproduce an image with the transmitted signal.

**Figure 25.**
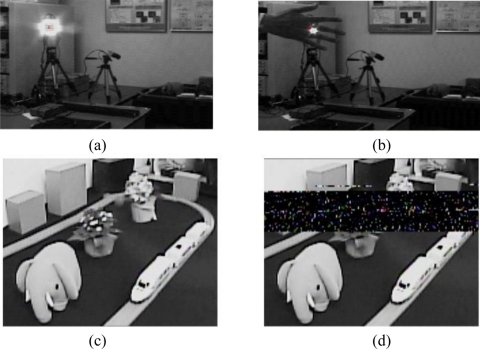
Image output of the prototype chip and reproduced images.

Figure 25(a) shows an image where an LED array is driven by the modulated signal of the captured image. Figure 25(c) is the reproduced image, which is transferred by the spatial optical communication. When the light communication pass is interrupted by hand as shown in Figure 25(b), errors in the reproduced image occur as shown in Figure 25(d). These results demonstrate that the proposed spatial optical communication system using the CMOS imager/LPR cell array successfully works.

In the experiment in Figure 25, the target light source is not moving. The final goal of the ISC chip is continuous communication for a moving target light source. To do this, investigations considering many aspects of the moving speed of the target light source and the ISC camera, the distance of communication, the sampling rate for the tracking and algorithm to use the 25-channel parallel LPR cell outputs are necessary, which are beyond the scope of the present paper.

## 6. Conclusions

In this paper, we have proposed and implemented a CMOS sensor for spatial optical communication of a long range of 100 m and moderate data rate of 1 Mbps. Using the CMOS sensor with 640 × 240 pixel array and 640 × 240 LPR cell array, a LED light of 1 MHz carrier frequency is successfully received. A proposed model of the transient response of the LPR cell agrees with the result of the device simulations and measurements. Spatial optical communication between a non-moving object (LED light source) and the non-moving receiver (ISC chip) has been demonstrated. The demonstration of optical communication for a moving target light source is left as a near future subject.

## Figures and Tables

**Table 1.** Summary of the simulation results.

Light Aperture Size		3 μm × 3 μm
Light Power Per Unit Area		0.49 W/cm^2^
Photocurrent		15.1 nA
Voltage Amplitude		82.3 mV
Time Constant	ON	14.0 ns
	OFF	14.2 ns
Node capacitance	ON	6.8 fF
	OFF	6.9 fF

**Table 2.** Parameters of fabricated sensor.

Process	0.18 μm CIS
Chip Size	8 mm × 7 mm
# of Imaging Pixels	640(H) × 240(V)
# of LPR Cells	640(H) × 240(V)
Pixel/LPR Cell Size	7.5 × 7.5 μm^2^
Fill Factor	20% (Imaging Pixel) 20% (LPR Cell)
Power Supply	3.3 V Analog 1.8 V Digital
Frame Rate	60 fps
Sensitivity	8.3 V/lx sec
